# Effect of Different Concentrations of Leukemia Inhibitory Factor on Gene Expression of Vascular Endothelial Growth Factor-A in Trophoblast Tumor Cell Line 

**DOI:** 10.22074/ijfs.2020.6058

**Published:** 2020-07-15

**Authors:** Khodakaram Jahanbin, Mehri Ghafourian, Mohammad Rashno

**Affiliations:** 1 Department of Immunology, School of Medicine, Ahvaz Jundishapur University of Medical Sciences, Ahvaz, Iran; 2 Fertility, Infertility, and Perinatology Research Center, Ahvaz Jundishapur University of Medical Sciences, Ahvaz, Iran; 3 Cellular and Molecular Research Center, Ahvaz Jundishapur University of Medical Sciences, Ahvaz, Iran

**Keywords:** Leukemia Inhibitory Factor, Trophoblast, Vascular Endothelial Growth Factor-A

## Abstract

**Background:**

Several studies have shown that leukemia inhibitory factor (LIF) is one of the most important cytokines participating in the process of embryo implantation and pregnancy, while, the role of this factor on vascular endothelial factor-A (VEGF-A), as one of the most important angiogenic factor, has not been fully investigated yet. The aim of this study was to evaluate the effect of LIF on gene expression of *VEGF* in the choriocarcinoma cells (JEG-3).

**Materials and Methods:**

In this experimental study, JEG-3 choriocarcinoma cells were treated with different concentrations of LIF (1, 10, and 50 ng) for 6, 12, 24, 48 and 72 hours. Expression of *VEGF* was analyzed by real-time PCR. Delta CTs were subjected to one-way analysis of variance (ANOVA) and a post hoc Tukey’s test by SPSS version 25.0 software for data analyzing.

**Results:**

In the stimulated cells, different concentrations of LIF caused significant decrease of *VEGF* gene expression (P<0.05) at 12, 24 and 48 hours. In contrast, it was increased after 72 hours (P<0.001). Analysis of data after 6 hours also showed that level of *VEGF* gene expression was significantly decreased by increasing LIF concentration (P<0.001).

**Conclusion:**

Expression level of *VEGF* gene was decreased in trophoblast cells (except after 72 hours) under the effectof different concentrations of LIF in a time-dependent manner. So, this study showed that further studies are needed to determine the effect of LIF on other angiogenic factors in trophoblast cells.

## Introduction

Leukemia inhibitory factor (LIF) is a glycoprotein cytokine with a molecular weight of 38-67 kDa. That is a member of the interleukin 6 family. LIF receptor is a heterodimer composed of two chains, gp130 and leukemia inhibitory factor receptor-β (LIFR-β) expressing on the surface of trophoblast cells (1, 2). LIF induce tyrosine phosphorylation in signal transducers and transcription factors of several trophoblast cell types, like choriocarcinoma cell line (JEG-3) (3, 4). Phosphorylation and signal transduction lead to migration, invasion, stimulation or suppression of various categories of genes in trophoblast cells (5, 6). Janus kinase 1 (JAK-1) and Signal transducer and activator of transcription 3 (STAT-3), play important roles in the signal transduction factors and activation of transcription in the LIF signaling (7, 8). VEGF is a homodimer glycoprotein which can stimulate angiogenesis and vasculogenesis by two types of its receptors like Fmslike tyrosine kinase 1 (Flt1) and kinase insert domain receptor (KDR) (9, 10). VEGF has many roles in early pregnancy, such as oocyte maturation and development, trophoblast proliferation, placenta angiogenesis, embryo implantation, maternal blood vessel growth and development of the embryonic blood vessels (11). Formation of the placenta in uterus depends on differentiation of extravillous cytotrophoblast (EVT) for invasion to the uterine stroma and forming endovascular trophoblast (12, 13). Incorrect differentiation of EVT cells leads to disruption of spiral artery remodeling, and this impairment in spiral artery remodeling can lead to preeclampsia and defective development of the fetus (13). Trophoblast invasion is a localized and temporary process. That is the main factor in the regulation of implantation and supply of oxygen to the fetus. By *VEGF* gene inactivation, invasion and migration of trophoblast cells are reduced (14). VEGF-A is one of the main factors of EVT differentiation to the endovascular trophoblast (15). Anti-angiogenic factors that reduce the amount of VEGF-A is one of the factors inhibiting formation of spiral arteries, which eventually associated with the creation of preeclampsia (16). VEGFA is one of the factors encoded by *VEGF* gene. Studies have shown that among all growth factors encoded by this gene, VEGF-A is the most potent type in stimulating angiogenesis (17). During formation of placenta, EVTs, involving in vascular reconstruction, acquire the features associated with epithelial cells, following the production of VEGF and its receptor expression on the surface (12, 18). These cells migrate to decidua, followed by replacement of the endothelial cells in the spiral arteries to form spiral arteries (19). In this study, a choriocarcinoma cell line JEG-3 (derived from fetal trophoblast tumor) was used as EVTs (20). This cell line has many biological and biochemical features of EVTs (Cells lining the blood vessels of villus in the placenta) (21). This cell line is able to produce progesterone, hCG, steroids and other hormones in the placenta (22). In this study we aimed to evaluate *VEGF* gene expression levels in trophoblast tumor cell line (JEG-3) at different times, while these cells were treated by different concentrations of LIF.

## Materials and Methods

This experimental study approved by Ethics Committee of Ahvaz Jundishapur University of Medical Sciences, Ahvaz, Iran (Ethics code: IR.AJUMS.REC.1395.577).

### Cell culture and treatment

JEG-3 choriocarcinoma cells were purchased from the Pasteur Institute of Iran (Tehran, Iran). These cells were maintained in Dulbecco’s modified Eagle’s medium-F12 (DMEM-F12; GIBCO, Ireland) supplemented with 10% heat-inactivated fetal bovine serum (FBS; GIBCO, Ireland) along with penicillin (BioIdea, Iran;100 units/ml) and streptomycin (BioIdea; 100μg/ml). All JEG-3 cultures were commenced at 106 cells/175-cm2 flask and maintained under standardized conditions (37˚C, 5% CO_2_, humidified atmosphere). The cells were trypsinized twice a week when confluence was estimated at over 75%. For all assays, JEG-3 cells were adjusted to 105 cells/ml. The cells (10^5^cell/ml) were seeded in six-well plates, following the resuspension in complete growth media. Before adding the stimuli, the cells were starved for 2 hours in medium without FBS. The cells were cultured per well in the presence and absence of different concentrations (1 ng/ml, 10 ng/ml, 50 ng/ml) (23, 24) of human LIF (Sigma-Aldrich, Germany), while non-stimulated cells were included as controls. Treated and non-treated cells were incubated for 3, 6, 12, 24, 48 and 72 hours at 37°C with 5% CO_2_. The cell culture supernatants were then collected by aspiration and centrifugation at 1000 g for 5 minutes and they were stored at -70°C until cytokine analysis. JEG-3 cells were harvested and kept at -70°C until total ribonucleic acid (RNA) extraction.

### Ribonucleic acid (RNA) isolation and real-time polymerase chain reaction (PCR) analysis

RNA was isolated using TRI Reagent (SinaClonCo., Iran). According to the manufacturer’s protocol, and the purity of extracted RNA was determined by the A260/ A280 ratio (A260/A280 ratio was 1.8). 50-100 ng RNA was reverse transcribed using cDNA synthesis kit (SinaClonCo.) and relative changes in *VEGF* mRNA level was quantified by real-time reverse transcription PCR (RT-PCR). Expression level of *VEGF* was determined by quantitative RT-PCR (qRT-PCR) using SYBR Green ® Premix Ex Taq (Takara, Japan) dye detection method on ABI StepOne PCR instrument (Applied Biosystems, USA), compared to *GAPDH* as an internal control. Initial denaturation at 95°C for 10 minutes, 40 cycles of annealing at 95°C for 15 seconds and extension at 68°C for 60 seconds. Rest 2009 and Excel software were used for the analysis of gene expression ratio. Gene-specific primers for *VEGF* and *GAPDH* are summarized in Table1. The fold change for target genes normalized by internal control was determined by the formula 2^-ΔΔCt^. All reactions were run in duplicate.

### Statistical analysis

All of the experiments were repeated in triplicates and data were demonstrated as means ± standard error (SE). Statistical software SPSS 25.0 and Graphpad Prism 8.0.1 were used for data analysis. Delta CTs were subjected to one-way ANOVA and a post hoc Tukey’s test, while the non-parametric Kruskal-Wallis test was used to compare the results of different experimental days. P values lower than 0.05 were considered statistically significant.

## Results

**Fig 1:**
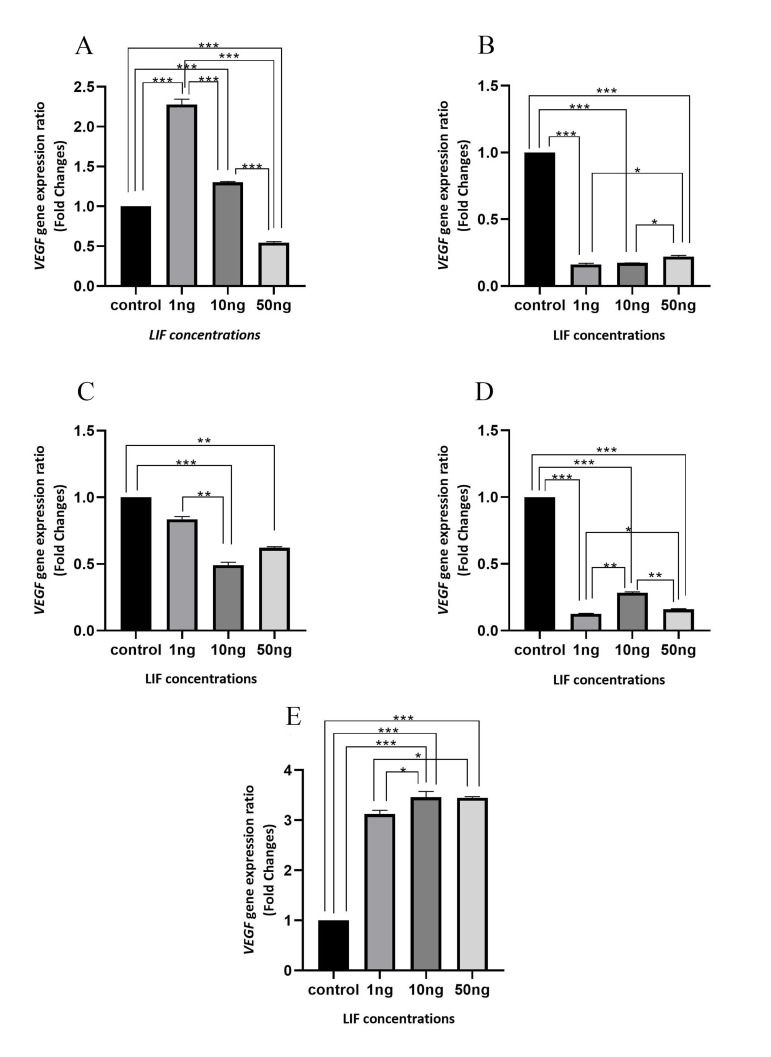
VEGF gene expression level at different time points, under treatment with different concentrations of LIF. The effect of different concentrations of LIF (1, 10 and 50 ng) on VEGF gene expression after **A.** 6 hours; **B.** 12 hours; **C.** 24 hours; **D.** 48 hours; and **E. **72 hours. Cells that did not treated by LIF were considered at any time as control, and the VEGF gene expression was measured in treated cells relative to these untreated cells. *; P<0.05, **; P≤0.01, and ***; P≤0.001

**Fig 2:**
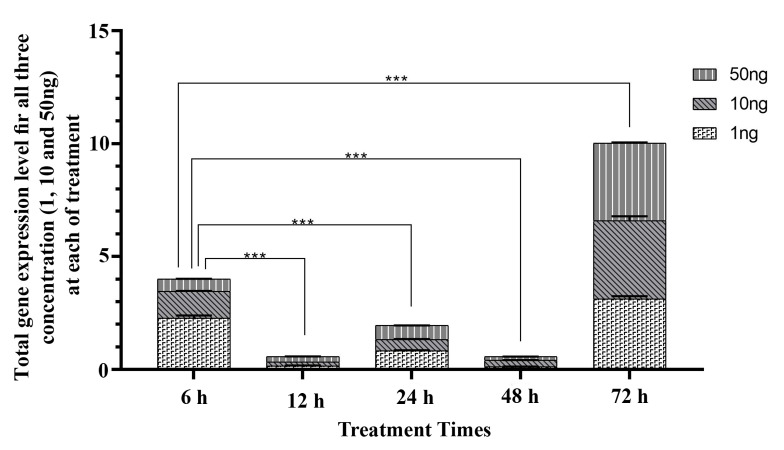
Comparing total VEGF gene expression at different time (6, 12, 24, 48 and 72 hours) under the treatment of different concentrations of LIF. *; P<0.05, **; P≤0.01, ***; P≤0.001) , and h; Hours.

Effects of different concentrations of LIF on VEGF gene expression level This study evaluated the effects of different concentrations of LIF on *VEGF* gene expression in different time periods, compared to untreated cells. The results are described (Fig .1, 2) in more details.

**Table 1:** Gene specific primers used for RT-PCR


Primer (accession)	Sequence (5'-3')	Tm	Amplicon size

*VEGF (NM_001287044.1)*	F: AGGAGGAGGGCAGAATCATCA	60	76 bp
	R: CTCGATTGGATGGCAGTAGCT		
*GAPDH (NM_002046.5)*	F: TGGGCTACACTGAGCACCAG	60	72 bp
	R: CAGCGTCAAAGGTGGAGGAG		


### Six hours treatment

An analysis of 6 hours data showed that by increasing LIF concentration, level of VEGF gene expression was decreased. In this time point, there is a significant difference (P<0.001) between the rate of VEGF gene expression in comparison with each other at different concentrations and control (Fig .1A).

### Twelve hours treatment

After 12 hours, there was a significant reduction in the *VEGF* gene expression in all three concentrations of LIF treatment than control (P<0.001). The lowest *VEGF* gene expression level was observed at 1 ng concentration of LIF. The results of 10 ng concentration of LIF were almost similar to the 1 ng (the difference between 1 and 10 ng was not significant). At 50 ng concentration of LIF, *VEGF* expression level was higher than the both concentrations of 1 and 10 ng (P<0.05, Fig .1B).

### Twenty-four hours treatment

Twenty-four hours after cells treatment with different concentrations of LIF, the results showed lowest expression of the *VEGF* gene at the concentration of 10 ng (P<0.001). Using 10 ng (P<0.001) and 50 ng (P<0.01) concentrations, there was a significant decrease in gene expression compared to control, but at 1 ng concentration, there was no significant decrease in the gene expression (P=0.324). Comparing gene expression between different concentrations of LIF showed a significant difference between the concentration of 1 ng and 10 ng (P=0.004, Fig .1C).

### Forty-eight hours treatment

After 48 hours, like 12 and 24 hours, *VEGF* gene expression was decreased by treating with different concentrations of LIF, compared to control (P<0.001), and the lowest gene expression was observed at 1 ng in comparison with 10 ng (P<0.01) and 50 ng (P<0.05). *VEGF* gene expression was more in 10 ng than the other two concentrations (1 and 50 ng) of LIF (P<0.01; Fig. 1D).

### Seventy-two hours treatment

After 72 hours, effect of LIF on the *VEGF* gene expression was reversed, and contrary to the previous times, in all three concentrations of LIF, we observed a dramatic increased expression of the *VEGF* gene, in comparison with control (P<0.001; Fig.2). The maximum *VEGF* gene expression was observed at 10 ng of LIF concentration, which had significant difference in comparison with 1 ng concentration of LIF (P<0.05). But, the difference between 50 ng and 10 ng LIF concentrations was not significant (Fig .1E).

### VEGF gene expression at different time points

**Fig 3:**
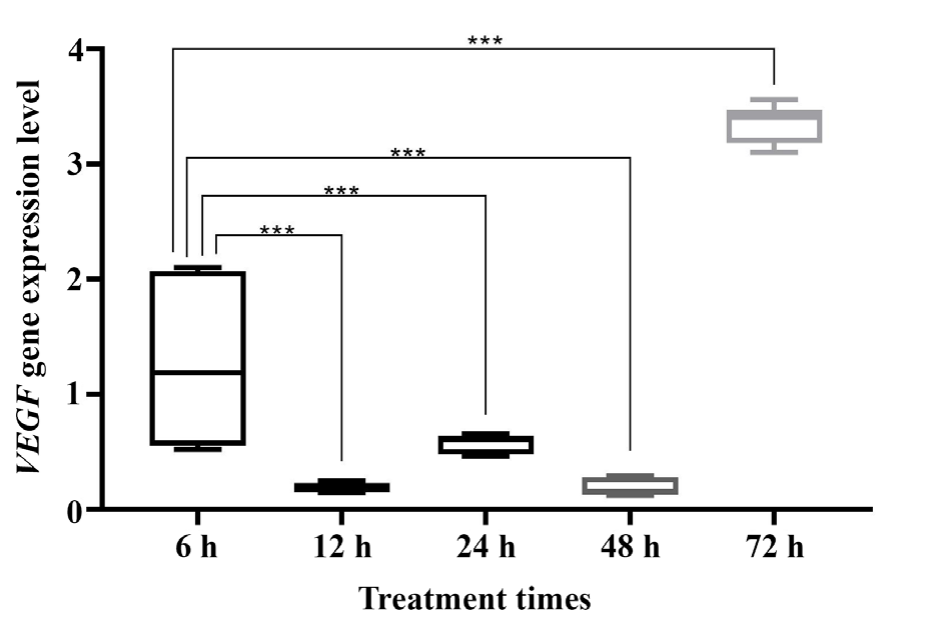
Changes in *VEGF* gene expression at different time points of inducting LIF to the cells. *; P<0.05, **; P≤0.01, ***; P≤0.001.

As shown in Figure 3, *VEGF* gene expression was dramatically decreased (P<0.001) at 12, 24, and 48 hours after cell treatment with LIF, in comparison with 6 hours treatment. In contrast to decrease in the *VEGF* gene expression at 12, 24 and 48 hours, we determined a significant increase (P<0.001) in *VEGF* gene expression at 72 hours compared to other time points.

## Discussion

Pregnancy is a complex process that depends on many factors. Studies have shown that cytokines, growth factors and several transcription factors play important roles in embryo implantation. For example, production of LIF by endometrial cells is essential for the beginning of implantation (25, 26). Data obtained from mice and humans have shown that among the all molecules expressed in uterus, LIF plays the most important role in embryo implantation (27). Formation of new blood vessels is called angiogenesis and it accompanies with migration, growth and differentiation of endothelial cells (28). Angiogenesis usually occurs during the menstrual cycle or estrus to convert the ovulation follicles to corpus luteum which leads to the synthesis of progesterone and restructure of the endometrium. This culminates in maintenance of embryo implantation (29). Angiogenesis and vasculogenesis are essential processes for increasing blood flow to the fetus and, consequently, supplying the nutrients and oxygen needed by fetus (13, 30, 31). Several growth factors control angiogenesis and vasculogenesis during pregnancy. Among these factors, VEGF plays a critical role in the development of the placenta and formation of vesJahanbin sels. Carmeliet et al. (32) showed that deactivation of onlyone VEGF allele leads to fetal death through angiogenesis disruption. Shalaby et al. (33) by disrupting Vascular endothelial growth factor receptor 1 (VEGFR1), Fong et al.(34) by disrupting VEGFR2 and Tsoi et al. (35) by disruption of neuropilin-1 and -2 (all of them are VEGF receptors) determined similar results to Carmeliet et al. (32). Adequate blood supply to the placenta is highly dependent on regulated invasion and trophoblast vascular remodeling in uterus (36, 37). Extravillous trophoblast (EVT) is a subset of trophoblasts that play the most important role in invasion (the same mechanism as cancerous cells for invasion) to the mother's uterus and vascular remodeling. This eventually acquires the phenotype of endothelial cells and improves artery formation (37). Previous studies have shown that EVTs have receptors for VEGF at their surface and message through these receptors which stimulate invasion, switch phenotype to endovascular cells and tube formation in EVT cells (37, 38). Defects in EVT invasion and angiogenesis have been observed in disorders, such as preeclampsia and intrauterine growth restriction (IUGR) (37). Due to the vital role of vascular formation by trophoblast cells (especially EVTs) in pregnancy and implantation, in this study, we decided to investigate the effect of LIF on one of the most important angiogenic factors, VEGF, in EVTs. For this purpose, we had to select an appropriate cell line with similar features to EVT cells. According to the previous studies (20, 39), JEG-3 cell line was selected. The results of this study showed that LIF could have a dual effect on *VEGF* gene expression with respect to time. So that at 12, 24, and 48 hours, *VEGF* gene expression was decreased, while it was increased at 6 and 72 hours (the increase of *VEGF* gene expression at 6 hours depended on the concentration of LIF showing a significant decrease at 50 ng concentration of LIF in contrast to 1 and 10 ng).

Considering the mentioned roles for VEGF during pregnancy and relevant disorders, as well as the important role of LIF during pregnancy, we decided to investigate the effect of LIF on the level of *VEGF* gene expression in trophoblast cells. Regarding to the results, it was found that the expression of the VEGF gene in trophoblast tumor cells treated by LIF was reduced in concentrationand time-dependent manners. Although expression of the *VEGF* gene was significantly increased after 72 hours, a study has previously shown that half-life of the LIF attachment to its receptor is slightly more than 24 hours (40). It can be concluded that after 72 hours, interactions between LIFs and their receptors are broken-down and the LIF signaling from their receptors are ended in trophoblast cells. As the results of this study showed. different concentrations of LIF can reduce the rate of *VEGF* gene expression depending on the time. So given the fact that *VEGF* gene expression level was decreased in LIF-treated cells, assessment of the production and secretion of VEGF protein in treated trophoblast cells is vital. Further investigations have to be performed on the other angiogenic factors to clarify the role of LIF on angiogenesis procedure in trophoblast cells.

## Conclusion

In conclusion, recent studies have shown that both LIF and VEGF are essential for maintaining and initiating the pregnancy process. It has also been found that angiogenesis process is a critical procedure in embryonic trophoblast cells for a normal pregnancy. VEGF-A is one of the most important angiogenic factors. Therefore, in this study we investigated the effect of LIF on *VEGF* gene expression in JEG-3 cell line as extravillous trophoblast cells. According to the results of this study, LIF causes a significant decrease in gene expression level of VEGF-A in JEG-3 cells. Further studies are needed to determine the action mechanism of LIF in angiogenesis of trophoblast cells.
